# Discovery urinary metabolomics of preterm neonatal acute kidney injury

**DOI:** 10.1007/s00467-026-07305-7

**Published:** 2026-05-05

**Authors:** Paige E. Condit, Nicole M. Nightingale, Benton J. Anderson, Ian J. Miller, Joshua J. Coon, Katherine A. Overmyer, Matthew W. Harer

**Affiliations:** 1 Division of Neonatology, Department of Pediatrics, University of Wisconsin School of Medicine and Public Health, Madison, WI USA; 2 Department of Biomolecular Chemistry, University of Wisconsin-Madison, Madison, WI 53706 USA; 3 Morgridge Institute for Research, Madison, WI 53515 USA; 4 Department of Chemistry, University of Wisconsin-Madison, Madison, WI 53706 USA

**Keywords:** Metabolomics, AKI, Neonates, Biomarkers, Noninvasive

## Abstract

**Background:**

Diagnosing acute kidney injury (AKI) in preterm neonates remains difficult, and its underlying causes are not well understood. This study aimed to compare urinary metabolite profiles in preterm neonates with and without AKI.

**Methods:**

We conducted a prospective observational study of neonates born at < 32 weeks’ gestation. AKI was staged using the modified neonatal definition. Urine samples were collected every six hours using cotton in diapers. Metabolomic profiling was performed via liquid chromatography mass spectrometry (LC–MS). Data were acquired in polarity switching mode and processed using Compound Discoverer 3.3.

**Results:**

Among 32 enrolled neonates (median birth weight 1.25 kg, gestational age 29 weeks), five developed AKI. A total of 987 urine samples were collected, with 522 age- and sex-matched samples from 16 individuals included in the final analysis. Principal component (PC) analysis revealed that PC1 and PC2 accounted for 15.82% and 11.61% of sample variance, respectively. Comparison of samples from neonates with and without AKI identified significantly elevated levels of furosemide, acesulfame, kynurenic acid, and hexaethylene glycol in the AKI group (*p* < 0.05, log2 fold change > 1) and with significant differences between metabolites within biochemical pathways including branch chain fatty acid oxidation, tyrosine metabolism, and carnitine synthesis. Longitudinal analysis also revealed metabolite changes preceding AKI onset, with distinct profiles compared to neonates who did not develop AKI.

**Conclusions:**

Preterm neonates with AKI exhibit distinct urinary metabolomic profiles compared to those without AKI. These findings suggest potential for metabolomic signatures to aid in early AKI detection, phenotype classification, and identification of therapeutic targets. Further research is needed to refine compound identification and timing of metabolite changes.

**Graphical abstract:**

**Figure:**
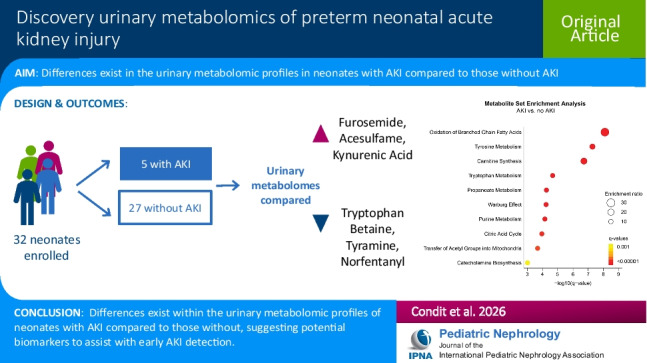
A higher resolution version of the Graphical abstract is available as Supplementary information

**Supplementary Information:**

The online version contains supplementary material available at 10.1007/s00467-026-07305-7.

## Introduction

Metabolomics is an emerging tool to define and detect clinical pathology with precision [1, 2]. With this technique, small molecules are identified and quantified in various biological components, including serum and urine, using various methods including liquid chromatography-mass spectrometry (LC–MS) [3]. The small molecules that are detected range from endogenous metabolites derived from cellular processes to endogenous compounds derived from food or drugs. Metabolomics is a valuable tool to measure kidney functional changes because the kidney is a very metabolically active organ, continuously filtering, secreting, and excreting many different molecules [4]. Furthermore, because the direct byproduct from kidneys — urine — is easily and noninvasively collected, longitudinal evaluations can be more easily achieved, potentially offering significant insight into disease trajectory [5–7].

The metabolomic methodology can be advantageous in identifying if differences exist between stressed and non-stressed states and whether changes in metabolites occur earlier or differently than standardly measured biomarkers [8]. For example, a previous study demonstrated the potential to distinguish differences in the urinary metabolome between preterm infants who developed surgical necrotizing enterocolitis versus those who did not [9]. However, additional studies evaluating differences in preterm infants who develop acute kidney injury (AKI) are lacking, and longitudinal collections that could inform about early diagnostic potential of metabolomics are needed.

To address this knowledge gap, we conducted a prospective study to evaluate the longitudinal urinary metabolomic profiles of preterm neonates and determine whether differences existed in the profiles between those who experienced AKI and those who did not. Our primary hypothesis was that there would be differences in the urinary metabolomic profiles in neonates with AKI compared to those without AKI.

## Methods

This prospective observational study was performed at the level III NICU at UnityPoint Health-Meriter Hospital (Madison, WI, USA) between April 2021 and June 2022. Institutional review board approval and informed consent were obtained from each participant's legal guardian before enrolling in the study (IRB #2017-013).

### Participants

This study was an arm of a prospective cohort study evaluating kidney injury and NIRS monitoring. Neonates born < 32 weeks’ gestational age (GA) were approached for informed parental consent. Exclusion of participants occurred if any of the following criteria were met: (1) enrollment and placement of NIRS sensors not possible by 96 h of life, which may have been due to the subject being deemed unsuitable for NIRS monitoring by the clinical team, or subjects could not be enrolled due to study staff unavailability, (2) non-English or non-Spanish speaking families, (3) mothers who could not participate in the consent process, (4) NIRS monitors unavailable, (5) documentation of congenital anomalies of the kidney or urinary tract. The reasons for unsuitability from the clinical team typically included compromised skin integrity (which would prevent proper sensor placement and signal acquisition) or severity of clinical illness that precluded additional monitoring.

### Outcome measures

The primary outcome measure of this study was to identify differences in metabolomic biomarkers between participants with and without AKI. AKI was defined by the modified neonatal KDIGO definition, including serum creatinine (sCr) and urine output (Supplemental Table 1) with baseline creatinine determined as the lowest creatinine after day of life two. All preterm neonates receive a basic metabolic profile (BMP) during their first 24 h of life, which includes sCr level to monitor for kidney injury and electrolyte imbalance. A second BMP is performed at 48–72 h of life and again at one week of age. If a preterm neonate is at risk of AKI within two to seven days of age, the team checks sCr levels more frequently. After one week of age, additional sCr are collected at the discretion of the attending physician. The neonate’s diapers are weighed every six hours to determine urine output until the neonate is clinically stable and off intravenous fluids.

### Urine collection

Urine was collected approximately every six hours by cotton in the participants’ diapers. Using a syringe, the urine was extracted into 0.5 mL plastic tubes in triplicate; one alioquot was reserved for LC–MS analysis. The samples were then immediately placed in a −  20 °C freezer. In the following 24 to 72 h, the samples were transferred to and stored in a −  80 °C freezer to prevent degradation.

Samples were prepared and LC–MS analysis was performed and processed [10, 11]. Additional information regarding sample preparation and analysis methodology can be referenced in the supplementary information.

### Demographic information

Basic demographic information including gestational age in weeks, sex, and birth weight in grams were collected. We also included the number of betamethasone doses received by the mother prior to delivery and the Apgar scores. We did not collect detailed information on medications or nutrition.

### Data analysis

The resulting data set was analyzed using Metaboanalyst 6.0 and with R statistical and plotting environment (v 4.4.0) [12]. To account for variations in dilution, individual sample data was normalized to total ion current (TIC) within the raw file and log2 transformed. Principal component analysis (PCA) was performed to transform the large data set into smaller sets while retaining data trends and variability. Urine samples were collected at six hour intervals, and retrospectively we assessed for the time of AKI and split the analysis into pre vs. post AKI. The effect of AKI was evaluated using linear regression models to account for confounding variables of sex and age. Significance was determined by model comparison of null model without the AKI variable using the anova() function, which returns the log-likelihood ratio and significance. Resulting p-values were adjusted for multiple hypothesis testing using the ‘fdr’ method in the p.adjust() function. Enrichment analysis was perfomed using Metaboanalyst 6.0 enrichment module based on quantitative values. The metabolite sets were derived from small molecule pathway database (SMPDB), and metabolite set analysis was performed with the detected metabolites as the reference [13].

## Results

**Fig. 1:**
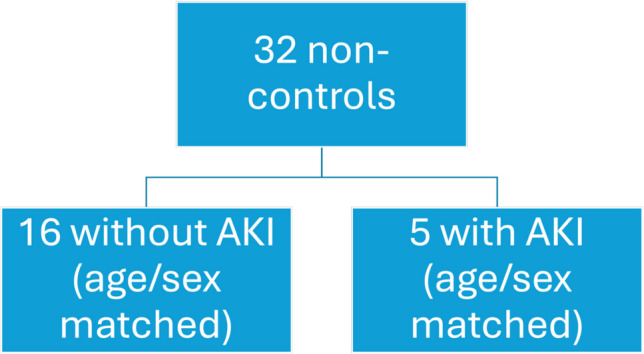
Consort diagram

Thirty-two patients were enrolled and included in the analysis (Fig. 1). Of the 32 participants included, five were diagnosed with AKI (13%). Of the five with AKI, four had stage 1, while one had stage 2, with a median day of life of AKI onset of 13 [IQR 7.5]. Two subjects were diagnosed with sCr criteria, two subjects were diagnosed with urine output criteria, and one subject met both sCr and urine output criteria for AKI diagnosis. The no AKI group had fewer creatinines checked compared to the AKI group (5 (IQR 3–8) vs. 11 (IQR 8.5–17.5), *p* = 0.005). Demographics of neonates with and without AKI can be seen in Table 1. Neonates with AKI were more likely to be of lower birth weight (850 vs. 1250 g, *p* < 0.01). To better match the sex and gestational age at birth between infants with AKI and no AKI, individuals were further separated into age- and sex-matched groups (*n* = 16, 5 AKI and 11 no AKI). Samples from the age and sex matched individuals were used for the downstream statistical analysis. On average we collected 30.8 samples per individual participant, and a total of 987 individual urine samples were analyzed by LC–MS.

**Table 1:** Demographics

	No AKI (*n* = 27)	AKI (*n* = 5)	*p*-value
Gestational age (wks), median (IQR)	29 (27–30)	27 (25–29)	0.13
Birth weight (g), median (IQR)	1250 (1020–1400)	870 (570–1075)	< 0.01
Sex, Female n (%)	13 (48)	3 (60)	0.63
1 min APGAR, median (IQR)	4 (5)	5 (3.5)	0.7
5 min APGAR, median (IQR)	8 (2)	8.5 (1.75)	0.16
Maternal bethamethasone prior to delivery (hours from first dose to birth), median (IQR)	33 (76.5)	70 (72)	0.82

**Fig. 2:**
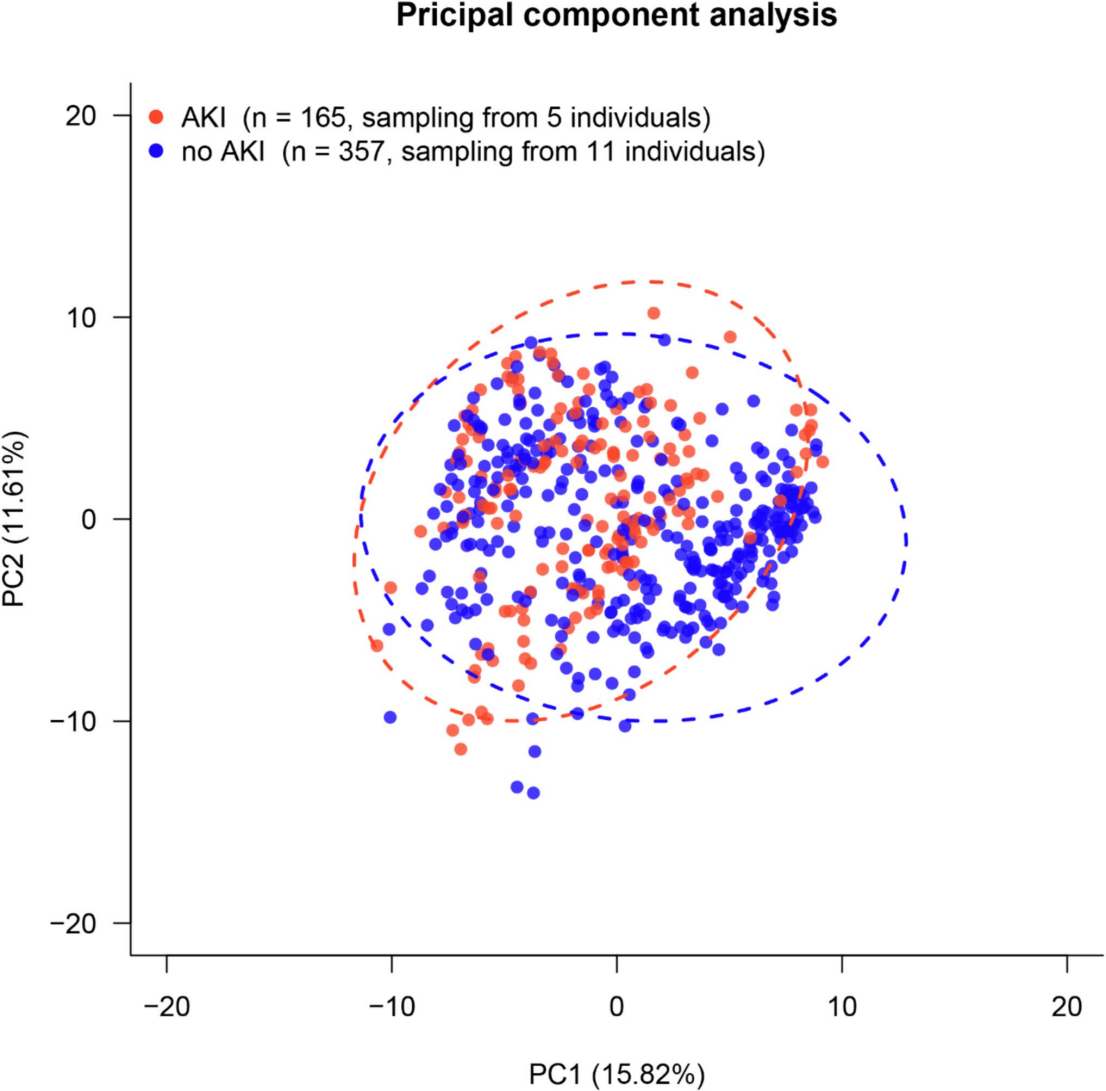
Principal Component Analysis. Neonates without AKI are represented by blue and the neonates with AKI are represented by red, with each individual point representing a composite of their metabolomic analysis and the blue ellipse or red ellipse representing the standard deviation

Initial assessment of the metabolomics data with a PCA demonstrates significant overlap between the AKI and no AKI groups (Fig. 2). Principal component 1 and principal component 2 capture 15.82% and 11.61% of variance between samples, respectively. Since together these two components only account for 27.43% of the variance in the data, this indicates significant heterogeneity in the samples.

**Fig. 3:**
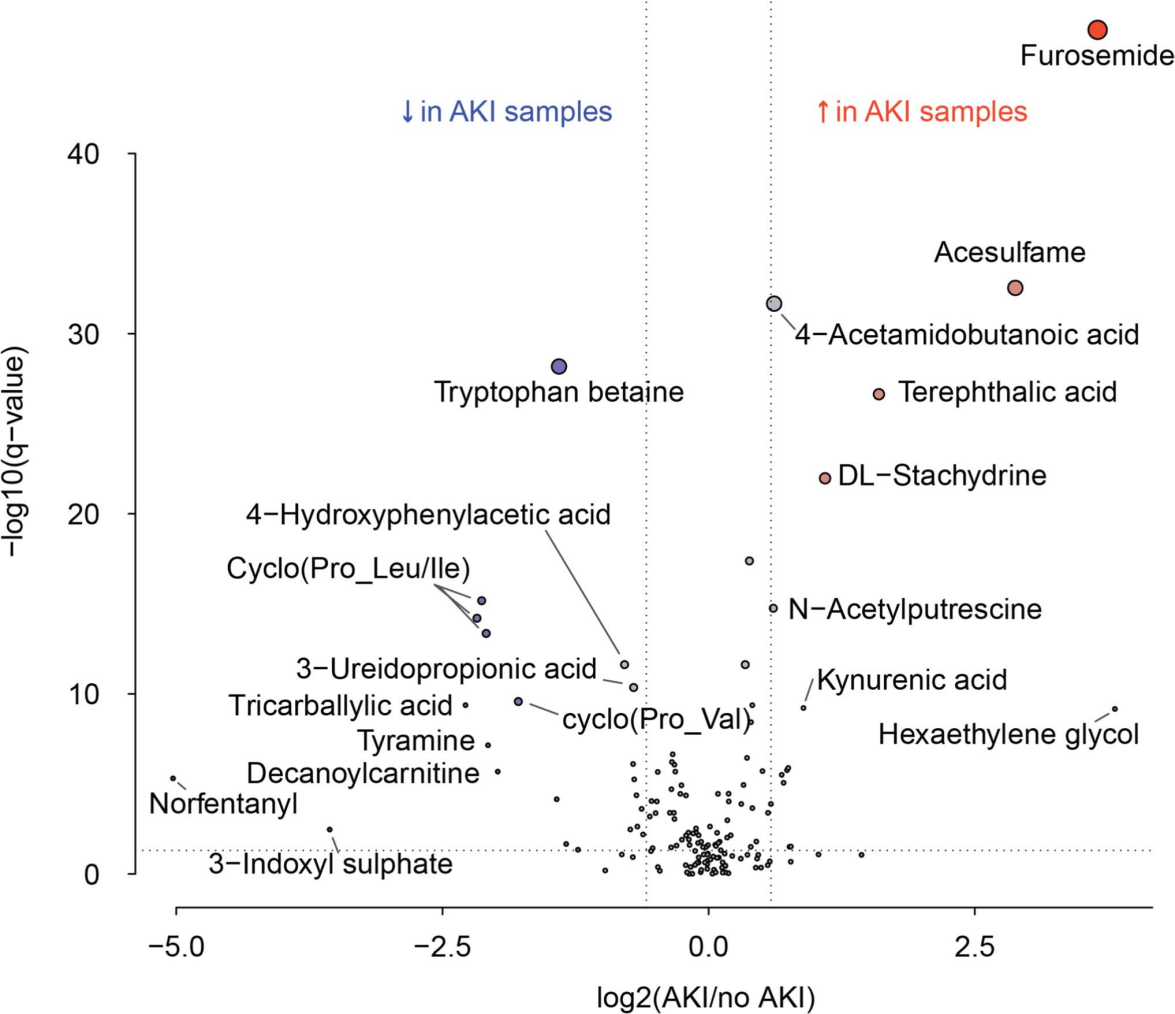
Volcano plot of all urinary metabolites by AKI status for age- and sex-matched AKI and no AKI groups. This volcano plot shows the fold change along the x axis and the statistical significance along the y axis. The points in red are metabolites that were increased in abundance in the AKI group and those with the blue marker were decreased in abundance in the AKI group

To determine if any metabolite abundances are significantly different between samples from AKI and no AKI groups, we performed a linear regession analysis including gestational age at birth and sex as confounding variables. The results from the linear regression analysis are visualized on a volcano plot, where differential abundance is shown on the x axis and significance of the effect is shown on the y axis (Fig. 3). Statistically significant elevations in furosemide, acesulfame, terephthalic acid, DL-stachydrine, kynurenic acid, and hexaethylene glycol are noted (Supplemental Table 2). Statistically significant decreases in tryptophan betaine, 4-hydroxyphenylacetic acid, cyclo (Pro_Leu/Ile), tricarballylic acid, tyramine, decanoylcarnitine, norfentanyl, and 3-indoxyl sulphate are noted (Supplemental Table 2).

**Fig. 4:**
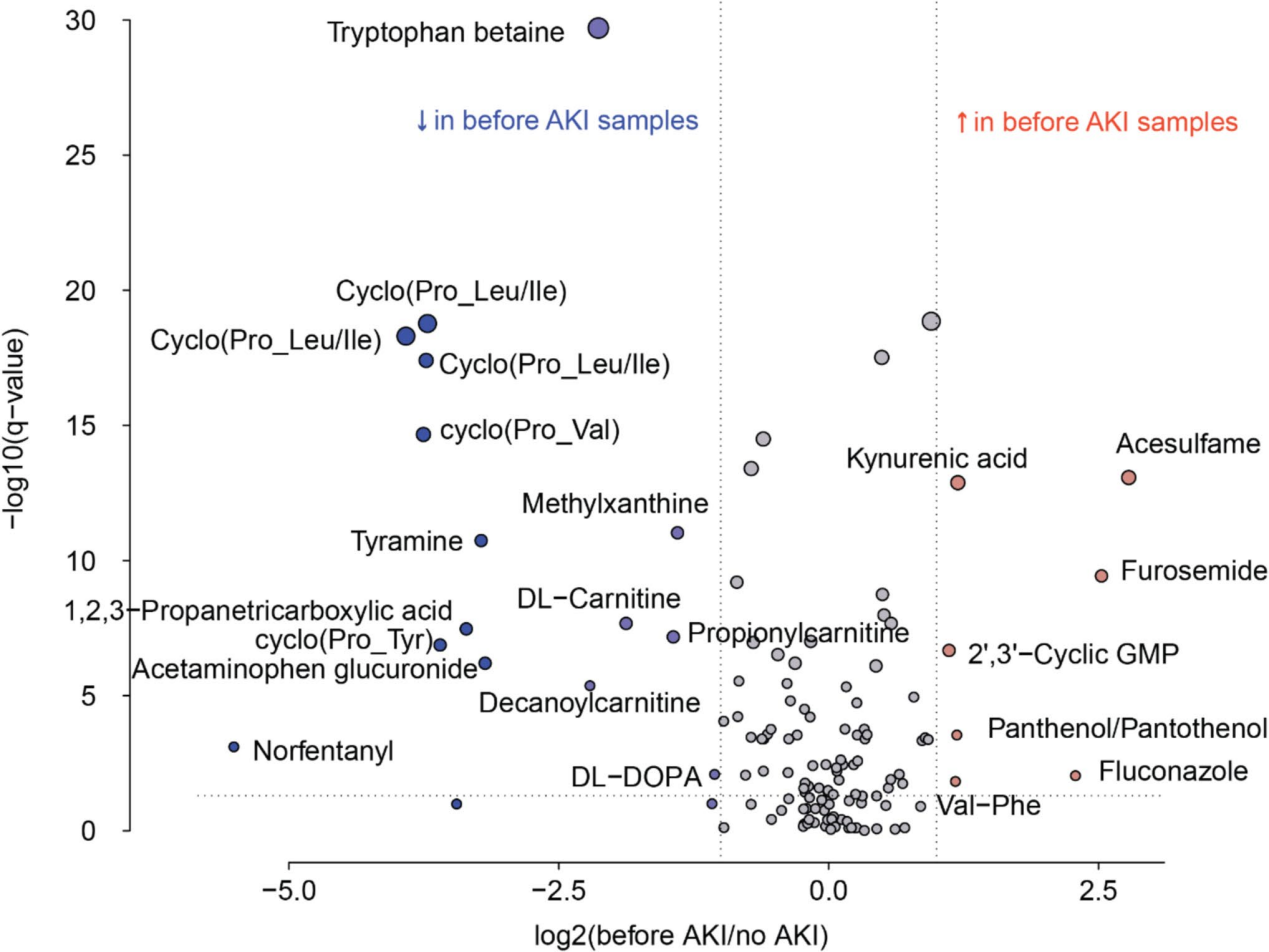
Volcano plot of metabolites by AKI status including only urinary samples prior to AKI for the AKI group, for all age- and sex-matched AKI and no AKI groups. This volcano plot shows the fold change along the x axis and the statistical significance along the y axis. The points in red are metabolites that were increased in abundance in the AKI group and those with the blue marker were decreased in abundance in the AKI group

**Fig. 5:**
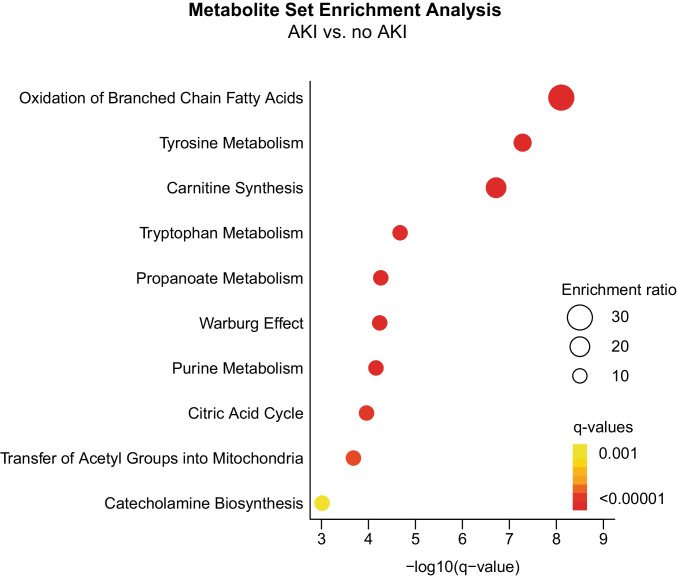
Enrichment analysis of metabolites most differentially abundant between AKI and no AKI samples. The top 10 most significant metabolite sets are presented with the circle size indicating the enrichment ratio

To determine if any metabolites are differentially abundant prior to AKI, we performed a linear regression specifically using data from urinary samples before AKI diagnosis, compared to the no AKI group, after adjusting for gestational age and sex. These data were visualized by a volcano plot (Fig. 4). Statistically significant elevations in acesulfame, kynurenic acid, furosemide, 2′3′ – cyclic GMP, panthenol/pantothenol, fluconazole, and Val-Phe are noted (Supplemental Table 3). Statistically significant decreases in tryptophan betaine, cyclo (Pro-Leu/Ile), cyclo (Pro-Val), methylxanthine, tyramine, DL-carnitine, 1,2,3-propanetricarboxylic acid, cylco (Pro-Tyr), acetaminophen, decanoylcarnitine, norfentanyl, and DL-DOPA are noted (Supplemental Table 3). Enrichment analysis shows that differentially abundant metabolites between AKI and no AKI samples were enriched in oxidation of branched chain fatty acids, tyrosine metabolism, and carnitine synthesis. The 10 pathways with the most significant enrichments are plotted in Fig. 5.

## Discussion

In this prospective study, we evaluated the differences in urinary metabolites in preterm neonates to assess whether there were differences between those who experienced AKI and those who did not. We found that significant differences exist between the urinary metabolomic profiles in neonates who experienced AKI versus those without, and those differences persisted after controlling for sex and gestational age. We also found that there were differences in the abundance of urinary metabolites before AKI onset compared to individuals who never experienced AKI, which could serve as potential biomarkers to improve precision in early AKI detection.

AKI in the NICU is common and follows a bimodal distribution for prevalence, affecting infants at the youngest and oldest gestational ages the most [14]. The AWAKEN study showed that even after controlling for numerous confounders, infants who experienced AKI had increased mortality. The current gold standard for AKI diagnosis is with the use of sCr; however, this marker often does not have detectable increases until there has been an approximately 50% decrease in kidney function. Currently there has not been a proven treatment option for neonatal AKI implemented based on sCr increases. If kidney injury could be detected earlier, before most of the kidney function is affected, perhaps there can be an effective therapy.

Few studies have evaluated the urinary metabolome of neonates, and none, to our knowledge, assessed metabolites over time. Mercier et al. evaluated urinary metabolites from day two of life with nuclear magnetic resonance (NMR) spectroscopy in very low birth weight neonates and found differences in the profiles of neonates who experienced AKI and those who did not [15]. In their study, they found differences in the urinary metabolites related to aminoacyl-t-RNA biosynthesis between neonates with and without AKI. This study could not further compare profiles before AKI to those without AKI, as urine was only collected at one time point.

Romick-Rosendale et al. also used NMR spectroscopy to profile the urinary metabolome in neonates and compared levels of carnitine between neonates with and without AKI [16]. Like our enrichment analysis, they found that urinary carnitine increased in neonates with AKI, and elevated urinary carnitine was more constant than other biomarkers, including neutrophil gelatinase-associated lipocalin (NGAL), making urinary carnitine a potential biomarker for neonatal AKI.

Our study used a discovery metabolomic approach to evaluate full spectra of components within the urine of neonates. This allowed us to identify potentially novel components, including those in the tryptophan metabolism pathway. Tryptophan 2,3-dioxygenase (TDO) and indoleamine 2,3-dioxygenase (IDO) metabolize tryptophan into kynurenine, which is further metabolized to kynurenic acid via kynurenine aminotransferases [17]. IDO expression is induced and enhanced by interferon-γ (IFN-γ) and tumor necrosis factor-α (TNF-α), two primary components of the innate immune system increased in states of inflammation, of which AKI may be considered [18]. Upregulation of IDO increased tryptophan breakdown, promoting the production of kynurenic acid. This is likely the mechanism by which urinary kynurenic acid is increased in our cohort. Previous animal studies reported increases in serum and urinary levels of kynurenic acid consistent with our findings [19–21]. Additionally, in adult ICU patients with AKI, urinary kynurenic acid levels were the most critical predictor of renal recovery on days 1 and 2 of AKI [22]. These findings have not been studied or replicated in neonates to our knowledge.

In our analysis, we discovered most notable significant differences in the metabolites in the branched chain fatty acid oxidation pathways, tyrosine metabolism, and carnitine synthesis, which not only may assist with detection of injury but also may allow for targeted therapeutic options.

Fatty acid oxidation, in concert with branched chain amino acid catabolism, is used in proximal tubule cells to generate ATP, and it is known that these pathways become downregulated in AKI, which likely explains the metabolite differences [23, 24]. Previous mice models have evaluated whether pharmacologic treatment with BT2 (3,6-dichlorobenzothiophene-2-carboxylic acid), a small-molecule which activates branched chain amino acid catabolism, in the setting of nephrotoxic AKI had improved outcomes and found that the mice with BT2 treatment had significantly protected kidney function [25]. Interestingly, there is an FDA approved drug prescribed for patients with urea cycle disorders, sodium phenylbutyrate, which binds to the same allosteric site and has the same mechanism of action as BT2 and could be potentially trialed in humans to determine whether kidney outcomes are improved in the setting of AKI [26, 27].

Tyrosine metabolism urinary metabolites are likely different because the kidney is one of the major sites where hydroxylation of phenylalanine occurs to synthesize tyrosine [28]. Previous work has shown that the conversation rate of phenylalanine to tyrosine is 50% lower in patients with kidney disease, and in mice with renal ischemia tyrosine levels were significantly reduced [29, 30]. While this pathway is an optimal target for biomarker development for AKI detection given the potential for high/low ratios on either side of the tyrosine synthesis pathway, it may also be possible to study whether the administration of BH4 (tetrahydrobiopterin) the cofactor for phenylalanine hydroxylase may protect against kidney injury as well. The pharmacological form of this molecule is sapropterin dihydrochloride and is currently approved for treatment of phenylketonuria [31]. Another possibility would be to study kidney outcomes with additional tyrosine supplementation.

The kidney is highly involved with carnitine synthesis as well as regulation, with the majority of carnitine filtered by the kidneys being reabsorbed [32, 33]. It would make sense with AKI that reabsorption is impaired to explain the urinary increases. Carnitine deficiency has been shown to increase dialysis-related complications in children and supplementation has been shown to improve kidney-related outcomes in patients with chronic kidney disease undergoing dialysis [34, 35]. This may serve as another area of study for AKI-related therapy beyond using the metabolite differences for potential biomarker development.

Reports of metabolomics in AKI have used NMR methodology more frequently, but use of LC–MS compared to NMR for metabolite detection and application in the clinical setting is faster, requires less sample for detection, and is more cost effective [36]. The use of this methodology in our study is more realistic for developing a tool in clinical applications; however, it has limitations, including more involved sample analysis and less quantitative results.

Our study is strengthened by its prospective nature with controlled urine collection timing, frequent urine sampling over time, and the ability to compare metabolites before and after AKI development. Limitations include the small sample size and single-center results.There also my be selection bias due to the clinical team determining adequacy for participation of the subjects and potentially having the most critically ill be excluded. Urine samples were collected with cotton placed in the diaper and it is possible the cotton may have affected the metabolomic results. However, all neonates had the urine collected in the same manner thus it is unlikely to have affected the differences seen between neonates with AKI and those without*.* Although several of the compounds significantly associated with AKI are clinicially administered drugs, we did not collect clinical medication records as part of the study and are unable to confirm administration dose or timing. Our findings will need to be replicated in a larger cohort across multiple sites to confirm that these metabolites are consistently elevated in neonates with AKI. Additionally, relationships with other more established AKI biomarkers including urinary NGAL should be explored to determine whether composite evaluations strengthen AKI detection and if there are differences between the measures.

In conclusion, determining a more sensitive and noninvasive biomarker for AKI in neonates could have a significant impact as it would allow for earlier detection and intervention. Given the high prevalence of morbidity and mortality associated with AKI in the NICU, finding a tool to detect injury when intervention may be more effective could significantly alter outcomes.

## Supplementary Information

Below is the link to the electronic supplementary material.

Supplementary file1 (DOCX 47.3 KB)

Graphical abstract (PPTX 158 KB)

## Data Availability

Data is available upon reasonable request, IRB approval, and proper data use agreement.
