# Supplementary material for: Discovery urinary metabolomics of preterm neonatal acute kidney injury
Source: Pediatr Nephrol. Author manuscript; Available in PMC 2026 May 18. (PMC13182702; doi:10.1007/s00467-026-07305-7)
Supplement: Supplementary Information [file NIHMS2175293-supplement-Supplementary_Information.docx]

**Supplementary Information**

**Supplemental Table 1**- KDIGO neonatal AKI diagnostic criteria

| **AKI stage** | **sCr criteria** | **Urine output criteria (hourly rate)** |
| --- | --- | --- |
| 0 | No change in sCr *or* sCr rise <0.3 mg/dL | ≥0.5 mL/kg/h |
| 1 | sCr rise ≥0.3 mg/dL rise within 48 h *or* sCr rise ≥1.5-1.9 $\times$ baseline sCr | <0.5 mL/kg/h $\times$ 6-12 h |
| 2 | sCr rise ≥2.0-2.9 $\times$ baseline sCr | <0.5 mL/kg/h for >12 h |
| 3 | sCr rise ≥ 3 $\times$ baseline sCr *or* sCr ≥ 2.5 mg/dL *or* kidney support utilization. | <0.3 mL/kg/h for ≥24 h *or* anuria for ≥12 h |

**Supplemental Table 2** – Significant metabolites discovered from analysis of AKI samples vs. no AKI samples, corrected for gestational age and sex. The number after the metabolite name indicates the LC-MS retention time.

| **Metabolite** | **Adjusted P-value** | **Log2(AKI/no AKI)** |
| --- | --- | --- |
| Hexaethylene glycol | 6.91E-10 | 3.815 |
| Furosemide | 1.36E-47 | 3.653 |
| Acesulfame | 2.88E-33 | 2.88 |
| Terephthalic acid | 2.31E-27 | 1.599 |
| DL-Stachydrine | 1.08E-22 | 1.093 |
| Kynurenic acid | 6.11E-10 | 0.89 |
| Hydroxyoctanoic acid, 6.265 | 3.01E-02 | 0.772 |
| Ala-Pro/Pro-Ala | 3.11E-02 | 0.76 |
| 3,5-Dimethoxybenzoic acid | 1.31E-06 | 0.747 |
| 2',3'-Cyclic GMP/3',5'-Cyclic GMP | 1.73E-06 | 0.736 |
| Phthalic acid | 8.72E-06 | 0.704 |
| Hippuric acid | 3.14E-06 | 0.686 |
| 4-Acetamidobutanoic acid | 2.16E-32 | 0.615 |
| N-Acetylputrescine | 1.77E-15 | 0.607 |
| Inosine | 1.31E-04 | 0.585 |
| Azelaic acid | 4.06E-04 | 0.557 |
| Glycocholic acid | 1.96E-06 | 0.506 |
| DL-Cystine | 3.70E-02 | -0.526 |
| Asp_Glu | 8.97E-05 | -0.537 |
| Ornithine | 6.47E-04 | -0.553 |
| DL-Glutamic acid | 6.44E-03 | -0.616 |
| 2-Furoylglycine | 2.43E-04 | -0.629 |
| Methylxanthine, 3.309 | 2.35E-03 | -0.669 |
| 2-Aminoadipic acid | 4.29E-05 | -0.678 |
| DL-Carnitine | 5.64E-06 | -0.699 |
| 3-Ureidopropionic acid | 4.46E-11 | -0.705 |
| Propionylcarnitine | 7.91E-07 | -0.71 |
| DL-Dopa | 3.39E-03 | -0.736 |
| 4-Hydroxyphenylacetic acid | 2.39E-12 | -0.79 |
| Metoclopramide | 4.53E-02 | -1.227 |
| Bilirubin | 2.18E-02 | -1.338 |
| Tryptophan betaine | 6.68E-29 | -1.405 |
| cyclo(Pro_Tyr) | 7.12E-05 | -1.427 |
| cyclo(Pro_Val) | 2.67E-10 | -1.787 |
| Decanoylcarnitine | 2.08E-06 | -1.981 |
| Tyramine | 7.14E-08 | -2.072 |
| Cyclo(Pro_Leu/Ile), 5.768 | 4.41E-14 | -2.089 |
| Cyclo(Pro_Leu/Ile), 5.645 | 6.74E-16 | -2.132 |
| Cyclo(Pro_Leu/Ile), 5.915 | 6.34E-15 | -2.176 |
| 1,2,3-Propanetricarboxylic acid | 4.25E-10 | -2.284 |
| 3-Indoxyl sulphate | 3.43E-03 | -3.559 |
| Norfentanyl | 4.97E-06 | -5.03 |

**Supplemental Table 3 -** Significant metabolites discovered from analysis with only pre-AKI urinary samples from the AKI group and all no-AKI samples corrected for gestational age and sex. The number after the metabolite name indicates the LC-MS retention time.

| **Metabolite** | **Adjusted P-value** | **Log2(before AKI/no AKI)** |
| --- | --- | --- |
| Acesulfame | 9.04E-14 | 2.78 |
| Furosemide | 3.73E-10 | 2.528 |
| Fluconazole | 9.17E-03 | 2.287 |
| Kynurenic acid | 1.41E-13 | 1.197 |
| Panthenol/Pantothenol | 2.78E-04 | 1.188 |
| Val-Phe | 1.49E-02 | 1.175 |
| 2',3'-Cyclic GMP/3',5'-Cyclic GMP | 2.10E-07 | 1.116 |
| DL-Stachydrine | 1.63E-19 | 0.95 |
| Terephthalic acid | 3.06E-09 | 0.931 |
| S-Adenosylmethionine | 4.19E-04 | 0.923 |
| Labetalol | 3.54E-04 | 0.894 |
| Azelaic acid | 4.60E-04 | 0.866 |
| Inosine | 1.05E-05 | 0.789 |
| Methylsuccinic acid | 1.37E-14 | 0.733 |
| N-Acetyl-L-tyrosine | 1.79E-02 | 0.686 |
| Ceftazidime | 8.10E-03 | 0.654 |
| N-Acetylputrescine | 2.01E-08 | 0.579 |
| Ampicillin | 1.26E-02 | 0.576 |
| 3,5-Dimethoxybenzoic acid | 2.62E-02 | 0.553 |
| Mandelic acid | 1.85E-06 | 0.54 |
| 4-Acetamidobutanoic acid | 9.65E-09 | 0.513 |
| Nicotinamide | 1.67E-04 | -0.535 |
| Citric acid | 2.56E-04 | -0.566 |
| 2-Furoic acid | 3.95E-04 | -0.6 |
| Theophylline | 6.13E-03 | -0.604 |
| Methylmalonic acid | 3.73E-15 | -0.606 |
| Sebacic acid | 2.36E-06 | -0.615 |
| Ornithine | 3.96E-04 | -0.618 |
| 3-Ureidopropionic acid | 1.05E-07 | -0.698 |
| Proline | 4.17E-14 | -0.717 |
| Methylxanthine, 3.509 | 3.37E-04 | -0.719 |
| Succinic acid | 8.69E-03 | -0.77 |
| 2-Aminoadipic acid | 3.81E-03 | -0.779 |
| Asp_Glu | 2.63E-06 | -0.832 |
| 2-Furoylglycine | 5.57E-05 | -0.842 |
| Pantothenic acid | 6.05E-10 | -0.85 |
| DL-Glutamic acid | 8.29E-05 | -0.972 |
| DL-Dopa | 8.15E-03 | -1.057 |
| 4-Hydroxyphenylacetic acid | 1.52E-13 | -1.082 |
| Methylxanthine, 3.309 | 9.24E-12 | -1.4 |
| Propionylcarnitine | 6.46E-08 | -1.439 |
| DL-Carnitine | 2.01E-08 | -1.876 |
| Tryptophan betaine | 2.28E-30 | -2.133 |
| Decanoylcarnitine | 3.95E-06 | -2.211 |
| Acetaminophen glucuronide | 6.15E-07 | -3.183 |
| Tyramine | 1.81E-11 | -3.219 |
| 1,2,3-Propanetricarboxylic acid | 3.25E-08 | -3.357 |
| cyclo(Pro_Tyr) | 1.32E-07 | -3.6 |
| Cyclo(Pro_Leu/Ile), 5.645 | 1.97E-19 | -3.716 |
| Cyclo(Pro_Leu/Ile), 5.768 | 4.60E-18 | -3.729 |
| cyclo(Pro_Val) | 2.53E-15 | -3.754 |
| Cyclo(Pro_Leu/Ile), 5.915 | 5.81E-19 | -3.916 |
| Norfentanyl | 7.82E-04 | -5.51 |

*Sample preparation*

All samples were maintained at -80 ̊C until the day of analysis. Due to the large number of samples present in the study, samples were analyzed in eight batches. To mitigate analytical bias in downstream analysis, samples were distributed into batches in a manner that ensured similar distributions of sex and gestational age for the no AKI subjects based on the AKI subjects demographics, then samples were randomized within batches to reduce bias further. Given the small number of patients overall and in the AKI group, batches were not exact matches of gestational age and sex. To limit the analysis time between batches and preserve sample integrity, each batch was prepared immediately before the end of the previous batch to keep the instrument running continuously throughout all eight batches.

Pooled urine samples was created by combining 14 unique neonatal samples. Briefly, the samples were thawed and combined, then centrifuged for 5 minutes at 1,500 × g at 4 ̊C and distributed into twelve 50 μL aliquots to serve as quality control samples. These pooled control alquots were stored at -80°C until time of study analysis. Additionally, a pooled urine dilution curve was prepared by diluting pooled urine with chilled water to 25% concentration, 50% concentration, and 75% concentration.

For each batch, samples and pooled urine were removed from the -80°C freezer and thawed at 4°C. Samples were then centrifuged for 1 min at 12,000 × g at 4 ̊C to remove any particulates. 50 μL of each sample was aliquoted into a glass-lined 96-well plate (Thermo Scientific, Part Number: 60180-P332). The samples and 96-well plate were maintained on ice before being placed into the autosampler for analysis. A silicone mat with slits (Thermo Scientific, Part Number: 60180-M112) was used to cover the wells to prevent evaporation. After LC-MS analysis, plates were sealed with foil covers (Research Products International, Part Number: ZC2007-24) and stored at -80°C.

*LC-MS Analysis*

Sample analysis was performed using a method previously reported [35]. Briefly, samples were separated using an Acquity UPLC HSS T3 column held at 40 °C (150 mm × 1 mm × 1.8 μm particle size; Waters). One μL of sample was injected on a Vanquish Neo System (Thermo Scientific) followed by separation with the following gradient at 70 μL/min flow rate: initial conditions of 100% Mobile phase A (0.1% formic acid in water) for 0.5 min, then linear increase to 100% Mobile phase B (0.1% formic acid in 95% methanol) over the next eight minutes. 100% Mobile phase B was maintained for 1 minute before returning to 0% mobile phase B over the next 0.75 minute and equilibrating at 0% Mobile phase B for the remaining 1.25 minute.

The LC system was coupled to a Q Exactive HF Orbitrap mass spectrometer through a heated electrospray ionization (HESI II) source (Thermo Scientific). Source conditions were as follows: HESI II probe at 30°C, capillary temperature at 300°C, sheath gas flow rate at 30 units, aux gas flow rate at 10 units, sweep gas flow rate at 1 units, spray voltage at |3.2 kV| for positive and negative mode, and S-lens RF at 50.0 units. The MS was operated in a polarity switching mode, acquiring positive and negative full MS and MS2 spectra (Top2) within the same injection. Acquisition parameters for full MS scans in both modes were 30,000 resolution, 1 × 10^6^ automatic gain control (AGC) target, 100 ms ion accumulation time (max IT), and 70 to 750 *m/z* scan range. MS2 scans in both modes were then performed at 30,000 resolution, 1 × 10^5^ AGC target, 50 ms max IT, 1.0 *m/z* isolation window, stepped normalized collision energy (NCE) at 20, 30, 40, and a 10.0 s dynamic exclusion.

*Data Processing*

The resulting LC-MS data were processed using Compound Discoverer 3.3 (Thermo Scientific). All peaks with a 0 min to 11.5 min retention time and 0 Da to 5000 Da MS1 precursor mass were aggregated into distinct chromatographic profiles (i.e., compound groups) using a 10-ppm mass and 0.5 min retention time tolerance. Profiles not reaching a minimum peak intensity of 1 ×10^6^, a maximum peak-width of 1, and a signal-to-noise (S/N) ratio of 3 were excluded from further processing. MS/MS spectra were searched within Compound Discoverer using mzCloud and mzVault spectral libraries. Annotations were assigned using a mass tolerance of 10 ppm. Data were filtered in Compound Discover, requiring each feature to be present in at least 100 of the samples with a Peak Rating Threshold of at least 5. Gap filling was performed with a mass tolerance of 10 ppm and an S/N threshold of 1.5. The resulting features were manually checked for quality, and putative identifications from the Compound Discover Compound table were used to annotate these features. Provided annotations are Level 1 or Level 2 annotations [36]. Metabolite features were removed if %RSD of replicate quality control was >30%.
